# Bacteriophages for Chronic Wound Treatment: From Traditional to Novel Delivery Systems

**DOI:** 10.3390/v12020235

**Published:** 2020-02-20

**Authors:** Ana M. Pinto, Miguel A. Cerqueira, Manuel Bañobre-Lópes, Lorenzo M. Pastrana, Sanna Sillankorva

**Affiliations:** 1 INL—International Iberian Nanotechnology Laboratory, Av. Mestre José Veiga, 4715-330 Braga, Portugal; mafalda.pinto@inl.int (A.M.P.); miguel.cerqueira@inl.int (M.A.C.); manuel.banobre@inl.int (M.B.-L.); lorenzo.pastrana@inl.int (L.M.P.); 2 CEB—Centre of Biological Engineering, LIBRO—Laboratório de Investigação em Biofilmes Rosário Oliveira, University of Minho, Campus de Gualtar, 4710-057 Braga, Portugal

**Keywords:** chronic wound, wound healing, biofilms, bacteriophage, phage therapy, delivery systems

## Abstract

The treatment and management of chronic wounds presents a massive financial burden for global health care systems, with significant and disturbing consequences for the patients affected. These wounds remain challenging to treat, reduce the patients’ life quality, and are responsible for a high percentage of limb amputations and many premature deaths. The presence of bacterial biofilms hampers chronic wound therapy due to the high tolerance of biofilm cells to many first- and second-line antibiotics. Due to the appearance of antibiotic-resistant and multidrug-resistant pathogens in these types of wounds, the research for alternative and complementary therapeutic approaches has increased. Bacteriophage (phage) therapy, discovered in the early 1900s, has been revived in the last few decades due to its antibacterial efficacy against antibiotic-resistant clinical isolates. Its use in the treatment of non-healing wounds has shown promising outcomes. In this review, we focus on the societal problems of chronic wounds, describe both the history and ongoing clinical trials of chronic wound-related treatments, and also outline experiments carried out for efficacy evaluation with different phage-host systems using *in vitro, ex vivo,* and *in vivo* animal models. We also describe the modern and most recent delivery systems developed for the incorporation of phages for species-targeted antibacterial control while protecting them upon exposure to harsh conditions, increasing the shelf life and facilitating storage of phage-based products. In this review, we also highlight the advances in phage therapy regulation.

## 1. Introduction

Chronic wounds are wounds that fail to progress in an orderly and timely set of stages of repair. These wounds have not reached the anatomic and functional integrity required over three months [1,2]. Often they will be vascular, diabetic, or pressure ulcers [3]. Non-healing wounds are a significant worldwide burden to health systems, affecting a substantial portion of the global population. These non-healing wounds are estimated to account for over 2.5 million people in the United States [4]. The prevalence of chronic wounds has increased due to the aging population combined with the increased rates of obesity in people and the consequent increased risk of developing diabetes [5,6]. These wounds cause significant patient morbidity, with adverse effects on the quality of life of patients and their families; increased pain that may lead to loss of function and mobility; distress, anxiety, and depression; and social isolation or even death [3].

Wound care standards focus primarily on identifying and removing the precipitating or aggravating factors to reduce inflammation and to enable the healing cascade to proceed [3]. Traditional chronic wound treatment strategies (e.g., compression, warming, vacuum-assisted closure devices, irrigation) are often successful in the healing of wounds, but many wounds have demonstrated recalcitrance to these treatments, leading to persistent and recurrent infections [5,7,8]. Often these treatments are also expensive and time-consuming [3,5] as these wounds remain open for long periods and their cost depends on the wound severity since this can involve more prolonged hospitalization and, consequently, more intensive care treatment [9]. The standard and widespread use of antibiotics has led to the spread of resistant bacteria. Bacterial resistance mechanisms are evolving and spreading globally, which threatens the current antibiotic treatment of infectious diseases [10]. The presence of antimicrobial-resistant pathogens in infections is a severe life-threatening condition that is increasingly challenging to treat [11]. Because of this enormous medical and economic burden, there is a need to develop therapies to overcome the current wound care healing barriers caused by the ineffectiveness of antibiotics in antimicrobial-resistant wound infections. Strategies, like bacteriophage therapy, have been suggested for bacterial infections.

This review provides an overview of chronic wounds, their economic burden, and incidence. It also focuses on the increase in the number of clinical trials using phages. Besides clinical phage therapy cases, this review also describes results from different types of models, reported after 2010, to understand the impact of phages on sessile cells and the new delivery systems developed for topical use in chronic wounds.

## 2. Impact of Chronic Wounds

Chronic wounds have increased significantly in prevalence in the last few decades, mostly due to the population aging and increases in the rates of obesity and diabetes which maintain wounds in a chronic low-level inflammation state, impairing healing [12]. Due to the different timeframes of healing per patient, which vary from four weeks up to more than three months [13,14], and the different nomenclatures used (e.g., chronic wounds, hard-to-heal wounds, difficult-to-heal wounds), the prevalence numbers are not the most accurate and vary according to the data collection model adopted [15]. These wounds are considered a global problem [16]. In addition, impaired wound healing affects millions of patients [4,17,18]. According to US estimates, chronic wounds affect 5–7 million patients each year, but international statistics are not easy to obtain [19]. Nevertheless, two studies about the burden of diseases, injuries, and risk factors were completed in 2015 and 2018 and included data from approximately 195 countries and territories, reporting an increase from 492 million (2005) to 605 million people (2015) and a decrease to 550 million in 2017 [20,21].

The direct medical cost of skin infections in the United States (US) is approximately 75 billion US dollars [22], with US$25 billion of this amount used for chronic wound treatment [23]. In the UK, the average cost of pressure ulcer treatments ranged from £1214 (category I ulcers, e.g., non-blanchable erythema) to £14,108 (category IV ulcers, e.g., full-thickness tissue loss with exposed muscle, tendon, or bone) [24].

**Figure 1:**
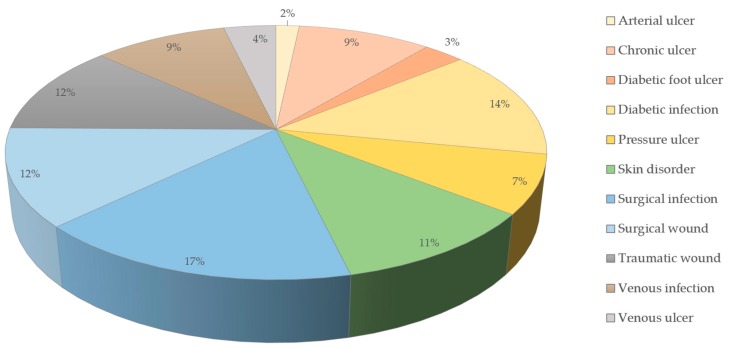
Prevalence of wound types in the Medicare population (data from the year 2014) [25].

A prevalence study from 2014 among the Medicare population in the US showed that at least one type of wound or wound-related infection was present in 14.5% of these patients. Surgical wound infections were the most prevalent (4.0%), followed by diabetic wound infections (3.4%) and non-healing surgical wounds (3.0%) [25] (Figure 1). Spendings ranged from US$28.1 to US$96.8 billion for all wound types. However, arterial and pressure ulcers were the ones requiring the most money for a single treatment [25].

## 3. Wound Healing

**Figure 2:**
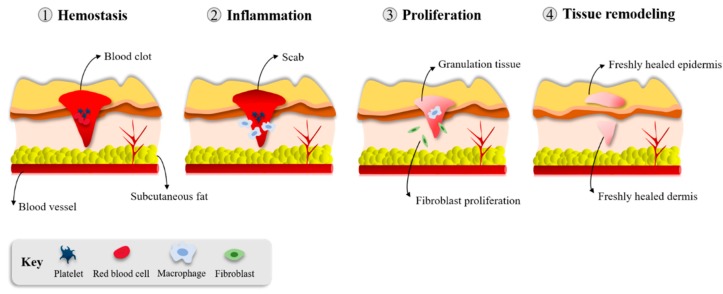
Phases of wound healing [4].

The burden of chronic wounds on health systems requires more work to investigate the basic science of wound healing and understand the conditions leading to these wounds [17,26]. The immune system plays an essential role in several repair mechanisms. Healthy skin wound healing takes place through a complex and delicate interaction of the immune system, keratinocytes, and dermal cells [17]. This healing process consists of four overlapping but distinct phases: hemostasis/inflammation, proliferation and repair, and, finally, tissue remodeling (Figure 2). These steps occur in a well-established sequence, at a specific moment, and continue at an optimal intensity for a particular duration [27].

### 3.1. Hemostasis/Inflammation

Hemostasis begins simultaneously with the inflammation phase, within the first minutes to hours, with the aggregation of plasma platelets forming a clot at the surface of the wound to prevent blood loss. The inflammation phase represents the initiation of the wound healing process, starting immediately after an injury has occurred through the migration of leukocytes to the wound site [4].

### 3.2. Proliferation and Repair

The proliferation phase begins after the inflammation phase has resolved. In this stage, vascular channels are re-established (angiogenesis), granulation tissue is generated (fibroplasia), and wound surfaces start to re-epithelialize [17,27]. The granulation tissue (comprised of type III collagen, fibroblasts, and new blood vessels) allows the formation of an epithelial barrier during epithelialization [4]. New blood vessels that enter the granulation tissue form to permit proper blood flow and the provision of wound healing factors [4]. Fibroblasts that acquire contractile properties can contract the wound edges and the migration and proliferation of keratinocytes, which are responsible for the closure of the lesion [17,27].

### 3.3. Tissue Remodeling

Remodeling is the last stage of the healing process and it starts several weeks after wounding and can take up to one year. The granulation tissue is remodeled by replacing collagen III by collagen I and there is a degradation of fibronectin and hyaluronic acid, forming a scar tissue rich in collagen fibers [17,27]. During this stage, the structural integrity and functional competence of the tissue restores and the maximum tensile strength is obtained [17,28]. Delayed wound healing and the possible formation of chronic ulcers, excessive scarring, or both can develop from dysregulation of either of these presented events [4,17]. In healthy individuals, this wound healing process is highly efficient, rapidly restoring the epidermal barrier functions [27].

## 4. Microorganisms Present in Chronic Wounds

**Figure 3:**
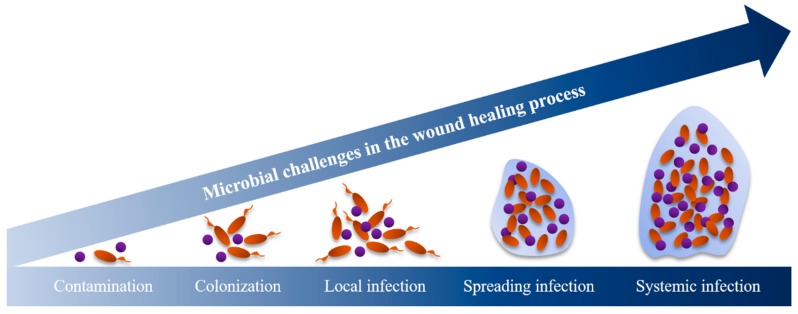
The wound infection continuum [18,30].

Bacteria have an essential role in hindering the healing process of chronic wounds. The proliferation of bacteria in wounds is often a concern as they can hinder the hosts’ immune response. Bacterial presence represents a continuum from contamination and colonization through to infection (Figure 3). When the organisms multiply, they interfere with wound healing and this tends to stagnate the healing process. After stagnation, denser bacterial biofilms lead to critical colonization. This step is commonly characterized by the presence of odor and discoloration without signs of infection (e.g., fever and inflammation) [29] and is followed by local infection. The surrounding tissues can also be infected, leading to deep infections or even systemic infections (Figure 3) [29].

The exposed subcutaneous tissue, devitalized tissue (ischemic, hypoxic, or necrotic), and the compromised immune system of the host provide optimal conditions for microbial colonization and growth [31]. Contamination by microorganisms frequently occurs from endogenous secretions, mainly through the transfer of healthy body bacteria of the surrounding skin [2,30]. For instance, *Staphylococcus epidermidis*, a commensal inhabitant of human skin and mucous membranes, has emerged as a relevant opportunistic pathogen in hospitals. This opportunistic pathogen causes infections, mainly in the elderly and immunocompromised individuals [32,33]. Wound contamination can also occur through cross-infection events, such as poor hand hygiene practiced by healthcare clinicians after wound cleansing and dressing procedures, coughing and sneezing, dirty bedding, unsterilized medical equipment, and prolonged use of catheters, tubes, or intravenous lines [34,35,36,37,38]. Wound contaminants can also derive from the environment. Exposure to unhygienic environments (presence of dust, unclean surfaces, mold/mildew in bathrooms) and prolonged hospitalization periods can be critical to patients with wounds [30]. These may increase the probability of wound contamination, including by antibiotic-resistant organisms, complicating wound treatment [30]. Therefore, it is vital to provide a suitable environment to allow wound healing and to avoid bacterial contamination. Some measures, such as performing wound care with appropriate aseptic techniques and in a clean environment, properly storing equipment and supplies, providing education for the patient and their caregivers, and acknowledging the current local policies and procedures, should be taken into account [30].

Microbial flora in chronic wounds appears to be complex and changes over time [2,39]. Aerobic or facultative pathogens (e.g., *Staphylococcus aureus*, *Pseudomonas aeruginosa*, and beta-hemolytic streptococci) are particularly prevalent in infections and delay wound healing [2,18,31,40]. In diabetic foot ulcers, *S. aureus* is the prevalent isolate together with others such as *P. aeruginosa*, *Enterococcus* spp., *Escherichia coli*, *Enterobacter* spp., *Proteus mirabilis,* and *Klebsiella pneumonia* [41,42,43]. In burn wounds, the presence of pathogens such as *P. aeruginosa* together with *K. pneumonia*, *Stenotrophomonas maltophilia,* and *Enterobacteriaceae* spp., and all multidrug-resistant (MDR) or even totally drug-resistant organisms can be deadly [18]. The leading cause of death of those burn injury patients who survive longer than 72 h is reported to be a microbial infection, causing 43% to 65% of mortality (reviewed in [44]). Infections caused by the pathogens led to sepsis, causing multi-organ failure, but other causes of mortality were reported, such as respiratory infections, cardiac arrest, and even brain death (e.g., neurological deterioration and cerebral stroke). In chronic venous leg ulcers, the prevalent bacteria are *S. aureus*, *Enterobacter faecalis*, *P. aeruginosa*, coagulase-negative staphylococci, *Proteus* spp., and anaerobic bacteria [39].

Tissue hypoxia or anoxia cause cell death and tissue necrosis in chronic wounds and create an ideal anaerobic environment for colonization, for instance, by *Prevotella*, *Bacteroides*, *Peptostreptococcus,* and *Porphyromonas* [2,31] that persist in these wounds for several days [31].

Different species of bacteria and fungi live on all the mucosal epithelial surfaces of the human body [45,46,47]. Besides bacterial multispecies biofilms, bacterial-fungal colonization can also be found in chronic wounds [48,49,50,51,52] where they can potentially form biofilms [18,50]. Biofilms resulting from bacterial-fungal interaction enhance resistance to antibiotics since the fungal hyphae provide a foundation where bacteria can attach and receive additional protection [18,53], further complicating the choice of a therapeutic approach. Patients with type 2 diabetes hospitalized due to infected lower-limb wounds presented with not only fungal species but also mixed bacterial-fungal flora [51]. The predominant fungal species reported were *Candida parapsilosis*, *Candida tropicalis*, and *Trichosporon asahii*, while *Enterococcus faecalis*, *S. aureus* and *P. aeruginosa* were the primary bacterial isolates. A previous cross-sectional study showed that up to 23% of chronic wounds contained fungi [54]. This study demonstrated that the most abundant fungi in polymicrobial infections belonged to the *Candida* genus as well as to *Curvularia*, *Malessezia*, *Aureobasidium*, *Cladosporium*, *Ulocladium*, *Engodontium,* and *Trichtophyton*. More recently, a 61-year old burn patient had *Aspergillus fumigatus,* which spread both on the healthy skin surface but also infiltrated the burn wounds [55]. According to the authors, the most probable hypothesis for this infection was that it occurred from the surrounding air.

Prolonged antibiotic exposure often causes the emergence of resistant organisms in these wounds, such as methicillin-resistant *S. aureus* (MRSA) or vancomycin-resistant enterococci [2].

## 5. Biofilms

Bacteria can aggregate together and form biofilms comprising of cells embedded within a microbially-produced matrix of extracellular polymeric substances (EPS) [56,57,58].

**Figure 4:**
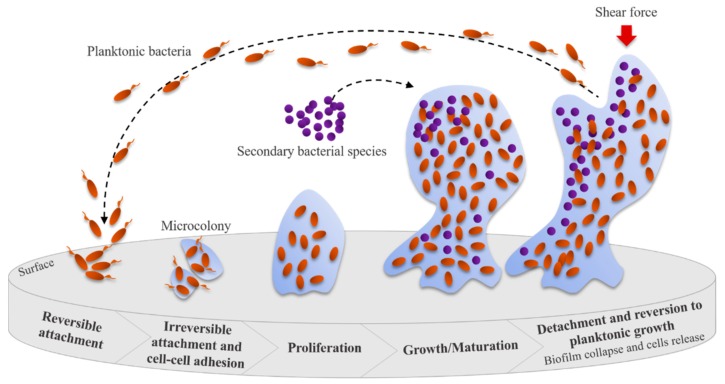
Steps leading to bacterial biofilm formation Adapted from [60].

Biofilm formation begins with a reversible attachment (intermediated by pili, flagella, or other surface appendages and receptors) of bacterial cells to a suitable surface (Figure 4). After that, an irreversible attachment occurs when bacteria start to grow and produce EPS that facilitate attachment and matrix formation. The biofilms continue to mature, often resulting in alterations in the phenotype of the microorganisms involved (e.g., growth rate and gene transcription changes), and induce biofilm growth in height or width. The collapse of the biofilm leads to an increase in bacterial cell motility within the matrix and their consequent dispersion. This event allows the attachment of these bacteria to other suitable surfaces, starting a new biofilm formation cycle [57,59]. The presence of microorganisms in biofilm communities leads to enhanced metabolic efficiency, accessibility to the substrate, higher resistance to stress and antibiotics, and increased capacity to infect and cause disease [56].

The biofilm structures vary according to the microbial species and their motility characteristics and can appear as flat biofilms with only a few layers, microcolonies, stalks, and multilayer mushroom-like structures [61,62]. These more complex structures frequently have water channels incorporated to allow liquid and oxygen flow, improving nutrient transport into cells, but also aid the delivery of antimicrobials to inner biofilm layers [63,64]. The population in biofilms is diverse and includes cells at different growth stages and also antimicrobial-resistant and persister cells [65]. The latter cells survive antimicrobial treatment due to their inactive metabolism which lacks protein synthesis [66,67,68], having reduced ATP levels [69] by remaining in a dormant state during the treatments. Persister cells remain viable and reproduce when the antimicrobial levels decrease and thus, are often responsible for the intractability of chronic infections [70]. Microorganisms in biofilms can also sense the density levels of other microorganisms in their proximity through an interconnected mechanism known as *quorum sensing*. *Quorum sensing* is a molecular/biochemical cell–cell communication mechanism mediated through the production of specific molecules that the cells excrete into the local environment. These molecules are sensed by the other local population, triggering, for instance, changes in the expression and regulation of genes, virulence, microbial competence, and also antibiotic resistance of the cells [71,72]. Also, the EPS matrix serves as a physical barrier to immune cells, limiting leukocyte and bactericidal product penetration and avoiding cell phagocytosis, resulting in collateral tissue damage and chronic wound inflammation; both of which delay healing [73]. Besides cell protection by the EPS matrix, biofilm cells also escape the immune system response by genetically activating the *quorum sense* response regulators, genetic switches, or suppressors [74]. Cell survival in biofilm communities profoundly challenges the treatment of biofilm-related infections, including the treatment of chronic wound infections [56,73].

In 2017, the World Health Organization (WHO) published a global priority list of antibiotic-resistant bacteria to help and guide research, discovery, and the development of new and effective antibiotics [75]. This list includes 12 species of bacteria categorized into critical (three species), high (six species), and medium (three species) according to their level of resistance. The critical species include *Acinetobacter baumannii* and *P. aeruginosa,* which are both resistant to carbapenems and *Enterobacteriaceae* (including *K. pneumonia*, *E. coli*, *Enterobacter* spp., *Serratia* spp., *Proteus* spp., and *Providencia* spp., *Morganella* spp.) carbapenem and third generation cephalosporin-resistant. The bacteria identified in the WHO list are well known for their ability to form biofilms [76,77,78,79]. For instance, *P. aeruginosa* possesses a high concentration of DNA within their EPS matrix, which promotes genetic variability inside the biofilm, increasing the probability of some individuals to resist changes in the environment (e.g., antibiotics) [80,81].

## 6. Non-Phage-Based Wound Treatments

For several thousand years, wound care merely focused on wound washing and application of plasters made of clay, plants, and herbs to absorb the wound exudate. Homemade dressings with honey, grease, and lint were also used to treat wounds and prevent their infection. Oil was also standard in wound care, both to inhibit bacterial growth and prevent plaster attachment to the wounds [4,29]. The first uses of honey date back to 3000 BC, with ancient Egyptian writings detailing its use in wound prescriptions [82]. Several factors combined are responsible for the antimicrobial properties of honey and these include the production of hydrogen peroxide, acidic pH, methylglyoxal levels, low availability of water, and high osmolarity, among others [83,84,85,86]. Due to the described properties, honey is still in use for wound treatment; however, not all types of honey have ideal properties and composition for antimicrobial wound treatment. The most well-known honey used in clinical applications is Manuka honey, which is available in many formats (e.g., ointments, adhesive bandages, dressings, pastes, gels) and commercialized for instance by Comvita (Medihoney products, Berkshire, UK), First Honey (Nashville, TN, US), and Advancis Medical (Activon products, Nottinghamshire, UK).

Later, treatments included dressings made of dry gauze or cotton wool, which required changing regularly due to the excess of exudate absorbed [4]. These played no role in the healing process, in contrast to more advanced dressings (e.g., semi-permeable films and foams, hydrogel, and hydrocolloid dressings (see review [87] for a more thorough discussion), which can improve healing through, for instance, the addition of active ingredients. A moist environment is crucial for wound healing, promoting keratinocyte migration [29]. Modern dressings are capable of both maintaining this moist wound environment and providing a physical barrier between the wound and contamination from the external environment [4,29]. Therapeutic outcomes and wound response to drugs can also be improved when dressings are fully dissolvable, non-replaceable, or non-adherent and if they distribute treatments (e.g., components that can help in the removal of necrotic tissues, prevent/treat infections, or both) to the wound site in a precise manner [88]. Wound dressings are, today, functionalized to incorporate several classes of antibiotics (e.g., quinolones, tetracyclines, aminoglycosides, cephalosporins) or other substances with antibacterial properties (e.g., essential oils) delivered directly at the wound sites [88]. The recruitment of cells, stimulation of cell proliferation, and regulation of extracellular matrix deposition can all be achieved using growth factors [4]. For instance, a dressing developed for drug delivery containing vascular endothelial growth factor, antibiotics, or both promoted angiogenesis, granulation tissue formation, and effectively controlled infection, speeding the wound healing and closure [89]. In brief, an optimal wound dressing must be able to protect the wound, maintain a moist environment, allow oxygenation, be non-adherent, antibacterial, cost-effective, and decrease the number of dressing changes [4]. Moreover, novel dressings must be appropriate for application during different stages of the wound healing process, always aiming for optimal function of the tissue [4].

Currently, an approach known as TIME, described by Schultz et al. [90], is used to treat acute and chronic wounds. Initially, debridement removes nonviable tissues (T). After that, infection and inflammation (I) are diminished by antibiotics and anti-inflammatory drugs and the moisture (M) is balanced using dressings. Finally, specific therapies to promote epithelialization (E) and the generation of new tissue are used [57].

Debridement can be accomplished surgically by mechanically removing necrotic tissue with scissors, a scalpel, or a curette under anesthesia, enzymatically using matrix-degrading enzymes (e.g., papain or collagenase) or biologically through debriding organisms such as medical-grade maggots [29,57]. This last technique is mostly reserved for recalcitrant fibrinous wounds but has demonstrated rapid and efficient necrotic tissue removal since the maggots’ saliva contains powerful enzymes that can dissolve the dead tissue [29].

The combination of cleansing, application of topical antimicrobials, or systemic antibiotics, in cases of deep infections, can improve wound healing [29]. For cleansing, normal saline or tap water can clean the wounds in contrast to irritating and toxic solutions (e.g., detergents, hydrogen peroxide, or concentrated povidone-iodine solution) that can cause additional tissue damage and cytotoxicity [13,29]. Topical antimicrobials provide direct targeting of the wound bacteria and, in contrast to systemic antimicrobials, are less likely to develop resistance among the colonizing population [29]. Nevertheless, bacterial resistance to topical agents can also occur and, once the wound is clean, the use of these agents should be discontinued [29]. Besides, topical antibiotics (e.g., gentamicin and neomycin) can induce hypersensitivity reactions and superinfections and thus, should be avoided [2,29]. Even though wound healing improvement is possible with cleansing agents and topical antimicrobials, for deep or systemic infections, systemic treatments are recommended [29]. For instance, systemic pentoxifylline, a xanthine derivative that decreases the viscosity of blood, improving its flow, healed venous leg ulcers compared to a placebo [91]. The only adverse effect observed was a gastrointestinal disturbance. Also, adding systemic granulocyte-colony stimulating factors, which help in the release and function improvement of neutrophil endothelial progenitor cells (usually lacking in diabetic patients), or hyperbaric oxygen, which improves the oxygen supply to wounds, showed a reduction in the need for more extended hospitalization stays and the need for amputations in people with chronic wounds [92,93]. Many other systemic drugs (e.g., aspirin, flavonoids, thromboxane alpha-2 agonists, sulodexide) improve the wound environment [94,95,96,97]. However, caution in the use of this latter treatment is needed to evaluate if the benefits overcome their associated risks: antimicrobial resistance, allergic reactions, drug toxicities (cardiac, hepatic, renal, and hematopoietic), and drug interactions [98].

Antiseptics are an alternative topical treatment for chronic wounds due to their microbicidal and broad antimicrobial spectrum of activity. They act on antibiotic-resistant microorganisms and reduce their development and have no or few systemic consequences when correctly applied [99]. The antiseptics that are currently in the market (e.g., iodine carriers with polyvinylpyrrolidone (povidone) iodine, silver-containing products, chlorhexidine, and dyes such as eosin [99]) are active and well-tolerated, a far cry from the early antiseptics that contained mercury- or arsenic-based compounds. Recent guidelines from a German–Austrian cooperation on the consensus regarding antiseptics recommend their use to prevent infection in wounds, decolonize wounds that present multi-drug resistant organisms, treat manifested infections, for use before debridement, and also for cleaning chronic wounds [100]. The criteria for opting for specific agents are not always straightforward, but primarily they have to have good efficacy towards the colonizing microorganisms and should kill above three logarithmic units, should not cause the development of resistance, be well tolerated and have no cytotoxic effects [101], should penetrate biofilms cells and necrotic tissue [102], and should decrease inflammation and improve healing [103]. Although no resistance to antiseptics from the peroxides/peroxy acids is known, some microbiostatic antiseptics show transferable resistances and can further be partially cross-resistant with certain antibiotics [104,105].

Besides these commonly used practices for wound management, new therapeutics have become more frequent and can even be used alone or complementary to systemic treatments. Some of the more recent therapeutic strategies include protein therapy (growth factors), gene therapy, platelet-rich plasma therapy, stem-cell-based therapy, and tissue engineering [106].

One of the recently revived therapies included in the novel therapeutics list is the use of bacteriophage (phage) therapy. Studies, mainly *in vitro, ex vivo,* and *in vivo* animal trials, have hugely increased in Western countries in the last few years, mostly due to the vast number of multi-drug resistant bacteria [106].

## 7. Phage Therapy

Phages, small viral entities that infect specific strains of bacteria, are abundant on our planet. They were discovered, independently, by Frederick William Twort (1915) and Félix d’Herelle (1917) [107,108]. Due to the immediate recognition of their potential as antibacterials, the treatment of many infections during the 1920s and 1930s was completed using phage therapy [108,109]. Even though Western countries abandoned this therapeutic approach, research continued in many Eastern European countries and the former Soviet Union [108,109]. Phage therapy uses include the treatment of many diseases from dysentery, cholera, and plague to skin infections, including wounds, and even respiratory tract infections [110]. The first documents related to phage efficacy are mostly in non-English languages and are not accessible for many readers (further reviewed in [110,111,112,113]). According to translated reports describing the unpublished results from d’Hérelle [114], phages were safe and effective in patients with dysentery and plague. Also, a French article dated 1921 described injection into and around skin infections eliminating the infection within 24 to 48 h [115]. Several results from the former USSR describe the successful use of phage therapy between 1922–1955, reporting mortality decreases and improved healing when administered as early as possible [116].

Phage therapy relies on the isolation and use of phages that exist naturally in the environment [117]. After isolation, phages are screened against frequently occurring pathogens, including drug-resistant and MDR bacteria, and evaluated through in vitro and in vivo models, including animal models and some human clinical trials [117,118]. Due to phages’ high specificity for their bacterial host, phage cocktail formulations usually guarantee a broader spectrum of activity. Also, the use of these mixtures targeting various receptors (e.g., lipopolysaccharides, type IV pilus, outer membrane proteins, etc.) decreases the likelihood of the emergence of phage-resistant bacterial mutants [117]. Furthermore, phage formulations can be applied alone or in combination with other antimicrobial agents (e.g., antibiotics) and their administration is possible using various routes (e.g., parenteral, oral, or local) [113].

### 7.1. Phage Therapy Reference Institutions

The Eliava Institute of Bacteriophages, Microbiology, and Virology and the Ludwik Hirszfeld Institute of Immunology and Experimental Therapy are reference institutions with an excellent clinical phage therapy background.

The Eliava Institute was founded in 1923 in Tbilisi, Georgia. At the Eliava Institute, phage therapy is used as a standard medical practice for prophylactic and treatment purposes in several hospitals and clinics and the absence of adverse effects has supported their clinical safety [107]. An example of a successful product, approved for commercialization in 2000 in Georgia, is PhageBioDerm, intended for the treatment of wounds. PhageBioDerm consists of a non-toxic, biodegradable polymeric material containing Pyophage cocktail, ciprofloxacin, benzocaine, α-chymotrypsin, and sodium bicarbonate [107,119]. The Pyophage cocktail formulation targets bacteria that are commonly present in purulent infections (e.g., *S. aureus*, *P. aeruginosa*, two *Proteus* species, *E. coli,* and several species of *Streptococcus*) [107]. Commercially-available Pyophage cocktails from two different manufacturers from Georgia and Russia revealed substantial differences in phage-types targeting the bacteria mentioned before, demonstrating the use of multiple strategies in their production [120]. These formulations have to be tested every six months, by law, against problematic strains to guarantee security and eventual upgrade by the addition of new phages [107].

The Hirszfeld Institute was founded in 1952 in Wrocław, Poland and has been actively involved in phage therapy since 1957. Their research focuses on the development and production of phages for the treatment of septicemia, furunculosis, pulmonary, and urinary tract infections and for the prophylaxis treatment of post-operative and post-traumatic infections [113]. Individual therapeutic phages have been used against MDR bacteria whenever these infections have resisted conventional antibiotics and were administered by local physicians [107].

### 7.2. Phage Therapy for Chronic Wound Healing—Ex Vivo and Animal In Vivo Models

Several recent studies have demonstrated the success of phage therapy in *ex vivo* and *in vivo* animal models and even in human patients. It is worth mentioning that phage replication *in vitro* is distinct from *in vivo* since several factors are impossible to replicate. Furthermore, *in vivo* processes change depending on the phage particle chosen.

*Ex vivo* models of wound infection and biofilm formation using porcine skin explants showed the effectiveness of phages against four pathogens commonly isolated from chronic wounds (*A. baumannii*, *P. aeruginosa, E. coli,* and *P. mirabilis*) [121]. In the porcine skin explant model, biofilms formed inside a skin cavity, closely mimicking a contaminated wound. In this model, the phage challenge was significantly more effective than in an *in vitro* model. A previous study by Oliveira et al. [122] showed that phages and chestnut honey action against biofilms formed in non-damaged porcine skin explants (which closely mimicked scratch wounds) increased with time against *E. coli* and *P. aeruginosa* mono and dual-species biofilms. The combined therapy resulted in bacterial reductions that were more effective on *E. coli* than on *P. aeruginosa* biofilms. The authors suggested that honey penetrated through the biofilm matrix, damaging the bacterial cell membrane and degrading the EPS matrix, further promoting phage infection of the biofilm cells.

In another work, another *ex vivo* porcine skin model of wound infection was used for monitoring bacterial growth, biofilm formation, and gene expression [123]. This model tested phage control of *S. aureus* biofilm formation and the population density-dependent regulation of virulence gene expression during *S. aureus* growth. The latter analysis evaluated the activity of the accessory gene regulator (*agr*) responsible for the control and regulation of gene expression in this strain [124]. Phage treatment caused a significant reduction of biofilm cells, confirmed by colorimetric assay of the tetrazolium salt XTT (sodium 3′-[1-[(phenylamino)-carbony]-3,4-tetrazolium]-bis(4-methoxy-6-nitro)benzene-sulfonic acid hydrate) and confocal laser scanning microscopy (CLSM), and quantitative real-time PCR (qRT-PCR) showed an increase in *agr*RNAIII expression during growth for most strains.

Phage therapy in animal models, such as in rodents and pigs, showed antimicrobial potential and wound healing capacity after the establishment of an infection with *S. aureus*, *P. aeruginosa*, and *A. baumannii* [125,126]. The efficacy of wild-type and biofilm-deficient strains of *S. aureus* tested with specific phages using a rabbit ear wound model showed considerable improvements in wound healing and biofilm cell reductions when a combination of phage therapy and surgical debridement was used [125]. Furthermore, biofilm-deficient *S. aureus* strains present in wounds significantly reduced in numbers and improved epithelialization and granulation of the wounds.

### 7.3. Clinical Phage Therapy Trials on Wounds

Clinical trials are mandatory before the official approval of therapeutics. Since 2007, phage therapy has been permitted for the treatment of bacterial infections in the Queen Astrid Military Hospital in Brussels, Belgium, under the umbrella of the Declaration of Helsinki (article §37) established by the World Medical Association. According to an article from 2018, external requests for phage therapy in this hospital have significantly increased in the last two years [127]. Phagoburn was a European Research & Development project funded by the European Commission in 2013 and its clinical trial (NCT02116010) ended in 2017. This trial aimed at evaluating the potential of phages in the treatment of burn wounds infected by the multi-drug resistant bacteria *E. coli* and *P. aeruginosa* [128]. The project was multicentered, involving 11 clinical partners from France, Belgium, and Switzerland, including the Military Health Service and Percy Military Hospital (France), the Royal Military Academy of Belgium, through the Queen Astrid Military Hospital and the Grand Hôpital de Charleroi-Loverval (Belgium), the Lausanne Burn Reference Centre (Switzerland), among others, and four SMEs were also involved (Pherecydes Pharma, Clean Cells, Statitec, and France Europe Innovation). The trial was a randomized phase 1/2 trial that recruited 26 patients with burn wound infections from nine burn centers from French and Belgian hospitals and one patient without infection and lasted 17 months in the years 2015 to 2017. A cocktail of 12 natural lytic anti-*P. aeruginosa* phages (PP1131, 1 × 10^6^ plaque forming units (PFU) per mL) was given to 13 patients topically for seven days and the wounds were followed for 14 additional days. The control group consisted of 13 patients receiving a topical standard of care cream (1% of sulfadiazine silver emulsion cream) using the same treatment regimen and one that did not receive treatment [128]. The bacterial loads in the infected burn wound patients receiving phage PP1131 decreased at a slower pace than in the group receiving the standard topical cream. The insufficient efficacy of the phage formulation on patients led to the early end of the clinical trial. The significant reasons for the reduced phage effectiveness were the emergence of resistant phenotypes and titer decrease after manufacture. The Phagoburn trial showed that only 10–100 PFU per mL were used and not the initially intended 1 × 10^6^ PFU/mL. The only positive outcome of the phage therapy was that it resulted in fewer adverse effects compared to the control treated group. The latter group showed increases in blood and lymphatic system disorders and infections other than wound infections (e.g., septic shock, bronchitis, pneumonia) [128].

Pherecydes Pharma is responsible for one ongoing clinical trial (PhagoPied, NCT02664740) carried out by the Centre Hospitalier Universitaire de Nīmes, France, aiming to evaluate the efficacy of standard treatment associated with topical application of a phage cocktail versus placebo for diabetic foot ulcers infected by MRSA. This study is not recruiting yet, but 60 adult participants (18 years and older) with no sex restriction, which have type 1 or type 2 diabetes and a wound below the ankle (evolving for >2weeks) mono-infected with MRSA will be included (https://clinicaltrials.gov).

One phase I clinical trial using phage therapy showed the treatment to be safe and efficient in chronic venous leg ulcers of 42 patients treated with *P. aeruginosa*, *S. aureus,* and *E. coli* phages [129].

A compassionate phage therapy treatment of nine patients with diabetes and toe ulcers infected by *S. aureus* (one MRSA and all other ulcers with methicillin-sensitive *S. aureus* (MSSA)) concluded recently. All patients admitted were unresponsive to conventional therapy between 10 days to seven weeks before phage treatment [130,131]. Staphylococcal phage Sb-1 topically applied to the ulcerations once a week, paired with standard wound care healed the ulcers in around seven weeks and severe ulcers in 18 weeks. Despite the poor vascularity and inadequate response to previous antibiotic treatment, the topical phage application successfully treated the *S. aureus* infected ulcerations. Furthermore, none of the patients’ files report adverse effects, tissue breakdown, or infection recurrences during or following treatment. After initiation of phage therapy, the inflammation resolved, the wound healed, and it eventually disappeared.

Another successful phage therapy study used Pyo bacteriophage preparation (NPO “Microgen”) for the treatment of diabetic foot ulcers in two MRSA colonized patients that healed and showed, in the end, no signs of MRSA [132].

Lytic Pyophage in sprays was common in Georgian soldiers’ first aid kits used on battlefields to minimize infection of inflicted wounds [107]. A small phase I study involving nine patients with *P. aeruginosa* infected burn wounds also used phages in spray solutions. In this study, wounds were sprayed once in a specific region, leaving a non-sprayed area of the wound to serve as a control. Tissue biopsies were removed before and after treatment from both areas [112]. By the end of the three week monitoring period of the burn wounds, patients showed no adverse effects and abnormalities due to the phages [112]. Nonetheless, spraying, for instance, of *S. aureus* phages showed a rapid decrease in viability and phages were no longer detected after 36–48 hours [133].

The effect of phage therapy was tested on 20 patients (aged between 12 and 60 years) with chronic nonhealing wounds (>six weeks) that did not respond to conventional local debridement and antibiotic treatment [134]. These wounds presented *E. coli*, *S. aureus*, and *P. aeruginosa* and 3 to 5 doses of the phage therapy led to complete healing or to healthy margins and healthy granulation tissue in the wounds.

AB-SA01, a phage preparation comprising three lytic *Myoviruses* phages with specific activity against *S. aureus,* was evaluated for its safety and tolerability in a clinical trial for topical administration (NCT02757755) [135]. AB-SA01 was safe and well-received by all patients that underwent administration. Several reviews provide an overview of other relevant studies using phage therapy against pathogenic bacteria present, for instance, in chronic rhinosinusitis, prosthetic valve endocarditis, and sepsis-associated inflammatory responses [136,137,138,139].

Despite all the studies, clinical trials, and literature reports, there are not yet any commercial phage products available for human therapy in the Western world. The non-commercialization of clinical phage formulations is mostly due to the complicated regulatory framework to accept their use [140], but recent discussions with regulating agencies have opened a new direction for regulatory approval [141]. Translation from preclinical phage therapy to human use beyond their current compassionate use under the guideline of the Helsinki Declaration is required.

## 8. Phage Delivery

The phage replication *in vivo* can only be predicted from their *in vitro* growth parameters to a limited extent [142,143]. Phage replication relies on several critical parameters related to the phage properties (e.g., adsorption rate, latency period, initial phage dosage) and the bacterial loads present. Additionally, *in vivo* administration of phages can depend on the selected time point and the delivery route, the phage particle clearance rate from body fluids, phage ability to replicate *in situ* including potential to form phage resistant bacterial variants, animal or human anatomophysiology, environmental conditions, and phage distribution in the human body, including specific effects of the immune system [144].

### 8.1. Individual Phages versus Cocktails

The application using a single phage has been a regular *in vitro* laboratory practice and also in most *in vivo* experiments. Nonetheless, after a few hours, there is an emergence of several phage resistant bacterial variants [145,146]. Susceptibility changes to the phages used can be due to the down-regulation, shielding, or modification of the host receptors that are essential for viral attachment or switching from a lysogenic to lytic cycle [147]. Overcoming phenotypic variations can be solved by combining different phages in a cocktail [145]. This approach permits not only a broader strain-specific range of such preparations, but can also decrease the emergence of resistant bacterial mutants and lead to a faster reduction of the number of bacteria compared to that of individual phage preparations [145,148,149].

A recently published guideline gathers some strategies to select the phages and their bacterial hosts for the development of an ideal phage cocktail [150]. According to the guideline, a phage cocktail formulation should be: (i) from a natural source (e.g., isolated from different environments such as water, sewage, soil, clinical samples, among others), without genetic manipulation; (ii) constituted of only lytic phages to avoid horizontal transfer of genes of potentially damaging genetic factors, and be well characterized; (iii) active against a broad range of target bacteria (e.g., it is sufficient if the phage lyses 70–80% of the target clinical isolates, but if an individual phage only infects 40–50% of the target strains, mixing with different phages enlarges this range); (iv) able to replicate on the target bacteria and have high rates of adsorption, a short latent period, and a large burst size; (v) constituted of phages with different bacterial cell wall receptor recognition sites to overcome potential bacterial resistance mechanisms; (vi) capable of maintaining the killing ability throughout treatments and storage. Phage-resistant mutants can arise, thus checking the frequency of bacterial resistant mutants (10^−7^–10^−8^ mutants per generation) is essential. For this, performing coculturing of the bacteria and phages in broth is sufficient. The phages must also remain stable, active over a long period of storage under different conditions and in different formulations (e.g., liquids, sprays, creams, gels, powders), and be compatible with other anti-infective agents. The authors of these guidelines draw attention to the fact that the mixtures should not be of phages randomly chosen since that combination can result in interference between them, causing even antagonistic effects. The Appelmans’ method for the assessment of the complementary activity of phages should be taken into consideration when selecting phages for the cocktail formulations [150]. This method compares the optical density (OD_600_) of a given bacterial strain infected with a phage mixture against the OD obtained after infection with the individual phages. When the OD values of mixtures are lower than the individual phage OD values, the phage mixture shows a positive outcome and, therefore, the phages can be combined. Also, the formulations used for therapy must be produced in a constant process of renovation and adjustment since new bacteria can arise from infection derived from different environments and geographic areas [150].

Furthermore, combined phage-antibiotic therapy is possible since bacterial cross-resistance to antibiotics and phages is unlikely to occur, with stronger resistances observed for antibacterial agents of the same type (antibiotic-antibiotic or phage-phage) [151].

### 8.2. Routes of Administration

The administration of phages can be through different delivery routes, including parenteral (intramuscular, intravenous, intraperitoneal), oral, and local phage delivery systems [152,153,154,155].

Polyclonal phage preparations are commonly inserted into wounds by different methods: (i) irrigation of the wound with a phage preparation after surgical debridement; (ii) ultrasonic debridement with the phage preparation; (iii) application of wound bandages impregnated with phage preparations; (iv) phage introduction periodically through drainage tubes; (v) application in film, powder, or drainage strip form [107,156]. Typically, phage preparations are used in local applications and injections one to three times a day for 3–7 days, according to age and wound issue nature [107,157]. The dose of wound preparations relies on the extent of the damage. Although direct injection of phages in the infection site reduces their possible loss, it is a more invasive strategy [119]. Topical application also reduces losses associated with absorption and distribution, increasing the antibacterial activity of phages [119].

### 8.3. Phage Delivery Systems

Lytic phages within topical solutions such as ointments, creams, and lotions can enhance the healing process of wounds [144,157]. These types of phage-containing solutions are easy to apply and remove (with soap and water only) and are stable throughout the treatment, which avoids the need for frequent applications and the use of bacteriostatic agents. Besides these characteristics, phages in the topical solutions have reduced adverse effects and very low toxicity for the patient [158]. In this type of vehicle, it is crucial to ensure proper incorporation of phages into the product so that a homogeneous and consistent distribution of the phages occurs during application to the wounds [157]. Topical phage and phage-based formulations alone have been useful in the treatment of skin diseases, but some factors can affect their delivery. For instance, the thicker ointment and paste formulations may have added components that can limit phage movement but also the presence of preservative agents in creams and ointments, particularly those with an acidic pH, can negatively impact the effectiveness of phages [159,160].

**Figure 5:**
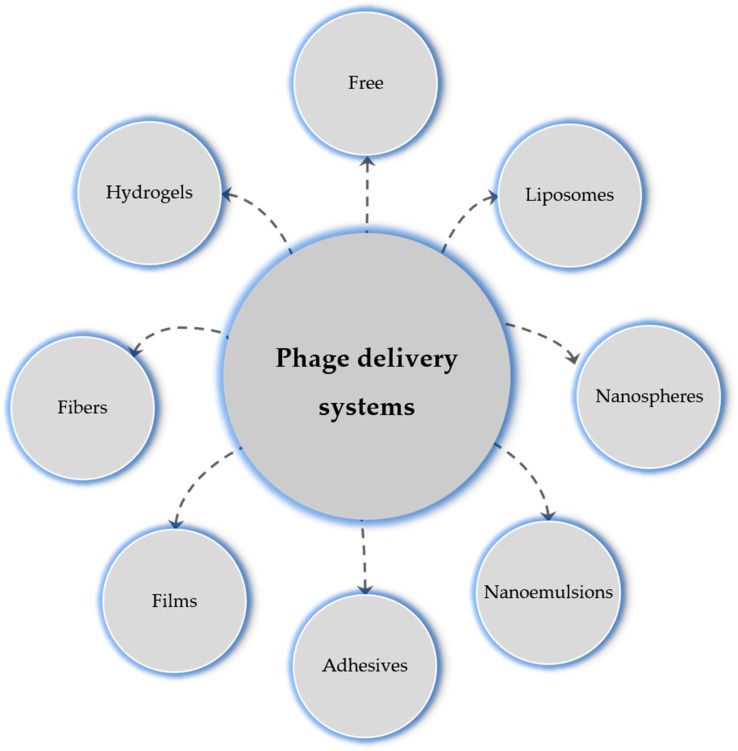
Phage delivery systems to wounds.

Many new delivery systems are now in vogue (Figure 5). Table 1 summarizes the different phage delivery strategies, models, and the targeted chronic wound microorganisms.

Incorporation of phages in different materials (e.g., polymers and lipids) allows the delivery of intact and viable phages to the desired destination [174]. For instance, phage encapsulation in liposomes, which are natural lipid vesicles, has been studied due to their biocompatibility with phages, biodegradability, non-toxicity, non-immunogenicity, and, perhaps most importantly, they are “Generally Recognized As Safe” (GRAS) [165,166,175]. Moreover, phage-loaded liposomes constitute a reservoir at the wound site, which releases phages at high concentrations during a significant period [117]. Chhibber et al. [166] evaluated the ability of a phage cocktail-loaded liposome to treat an *S. aureus*-induced diabetic excision wound infection. This study concluded that liposomal entrapment of a phage cocktail led to more available viable phages and a better phage persistence at the wound sites. Increases in phage titers and rates of infection resolution and wound healing occurred more rapidly when compared to the non-encapsulated free phage cocktail. These FDA approved liposomes have some disadvantages, such as degrading quickly *in vivo*, and their large size can affect their penetration and diffusion [176].

Different hydrophilic and hydrophobic polymers can also be used [174]. The most common are agarose, cellulose, alginate, chitosan, pectin, poly(dl-lactide: glycolide), Polyvinyl alcohol (PVA), Polyvinyl pyrrolidone, hydroxypropyl methylcellulose, hyaluronic acid methacrylate, among others [117]. A recent study used a novel PVA-Sodium alginate hydrogel-based dressing to deliver phage MR10 together with minocycline to burn wounds topically. *In vivo* experiments reduced bacterial colonization and wound contraction and further reduced inflammation in murine models with burn wounds infected by MRSA. Also, the combined treatment proved to be better than phage and antibiotic alone [173].

The use of biocompatible polyesters for phage encapsulation relies on their ability to provide mechanical strength through the reinforcement of hemostatic materials [171]. Polycaprolactone and collagen I nanofibers incorporating phage T4 produced by electrospinning eradicated *E. coli* infections and allowed hemostasis. The *in vivo* experiment of these nanofibers in rabbits demonstrated that the membrane was fully degraded in eight weeks and had an excellent antibacterial efficacy [171].

## 9. Phage Therapy Regulation

### 9.1. Regulation Hurdles

Phage therapy faces regulations and policy issues have not been favorable for its clinical use despite the many successful trials [137]. Modern drug regulation, implemented to avoid the deformities and deaths witnessed in the mid-20th century due to medicines containing, for example, diethylene glycol and thalidomide [177], reshaped the regulatory systems in many countries. For instance, through the formation of a Committee on the Safety of Drugs (the UK, 1963) or when the US Drug Amendments Act, passed by the Congress in 1962, started demanding FDA approval for all new drug applications to verify their safety, effectiveness, and compliance with good manufacturing practice (GMP) conditions. In the European Community, regulatory frameshifts came to use in 1975 through two council directives. These later led to the establishment of the European Medicines Evaluation Agency in 1993. After the initial reshaping period, harmonization of technical requirements occurred regionally, inter-regionally, and internationally. This modern pharmaceutical legislation has never been favorable for phage therapeutics and is a key reason for their non-approval [178,179,180]. The main reasons why phages do not yet have a legal framework include: (i) the complicated legal processes for the application of phage therapy that diverge between countries [181]; (ii) the diversity of phages and their characteristics that do not fit the standard regulation practices; (iii) the concerns regarding their production, purification, and cocktail formulation, which can vary tremendously to provide a patient-targeted therapy, and loss of phage viability from manufacture to being delivered to the patient; (iv) the differences between the action of antibiotics and phages since the latter are self-reproducing and increase in concentrations upon killing the target host, while antibiotics decline in concentration due to the patient’s metabolism and elimination by both hepatic and renal mechanisms; (v) the evolution that phages undergo, although at lower probabilities than the emergence of phage-resistant bacterial phenotypes; and (vi) the inconsistent treatment outcome that can vary from patient to patient, from success to complete failure if not given appropriately.

### 9.2. Recent Regulatory Decisions Regarding the Use of Phages

While the Georgian and Russian healthcare systems include phages as a standard medical application, legal and regulatory frameworks that define phages in the therapeutic context for human use are not yet found anywhere else [137,182]. However, compassionate therapy under article §37 of the Helsinki Declaration as an experimental therapy is allowed according to the World Medical Association [108,183]. Recently, the FDA has expanded schemes of non-approved therapeutics, including compassionate phage therapy use in patients as an emergency investigational new drug, such as when no comparable or satisfactory alternative therapy options are available or when the patient has an immediately life-threatening disease or condition [184]. The French National Agency for Medicines and Health Products Safety has also issued a temporary use authorization for compassionate therapy as long as the application for its use details information from clinical trials (particularly safety and efficacy, ensuring a positive benefit–risk ratio), describes the patient or groups of patients receiving therapy, and justifies its need and absence of an alternative approach [185]. In Australia, the Therapeutics Goods Administration have described in their regulations several ways that allow patients to gain access to products that have not been approved [186]. These are also always on a patient-to-patient basis and the use must be prescribed by medical practitioners that have the authority to prescribe unapproved therapeutic goods, such as phage products. However, in Australia, the patient can also import unapproved therapeutic goods for personal use but, in these cases, they should be aware that the quality, safety, and efficacy are unknown and any risk and adverse effects associated with their use are of their own responsibility [187]. In Belgium, while the Federal Agency for Medicines and Health Products granted permission to use phages as ingredients of magistral preparations [188], these have to comply with the requirements of the European Pharmacopoeia, of the Belgian Pharmacopoeia, or an official pharmacopeia [189]. Although in France the phage treatment is 100% reimbursed by the National Health Insurance, there is uncertainty in many other countries whether the costs are the responsibility of the provider, the patient, or their insurance company [181].

### 9.3. Prospective Future Issues for Use

A recent review summarizes the main issues that can influence the future development of phage therapy [190]. These include the regulatory/ethical/awareness-raising questions, which subsequently lead to difficulties in assembling optimal phage preparations that can become a promising alternative or complementary therapy to antibiotics [137,141,191].

For the success of phage therapy and to delineate clinical guidelines, crucial information and robust clinical trial data are needed. In 2017, Abedon described the different data that phage therapy researchers should report [192]. These include: (i) appropriate characterization and selection of phages, including source, characteristics such as burst size, latent period, absorption rates, bioinformatical characterization (GenBank accession number for phages with completed sequences), and justification to the use of a specific phage and no other; (ii) characterization and selection of the individuals submitted to the therapy; (iii) characterization and selection of the target bacteria, including nucleotide database and culture collection accession numbers or both, relevant descriptive publications, and antibiotic resistance characteristics; (iv) information about the formulations, dosages, and efficacy; (v) information about the routes of administration chosen (e.g., topical, injection, ingestion, through aerosols). These considerations are essential for the quality of future research, enabling researchers to replicate and extend previous studies, and can help future clinical applications [137,192].

One of the main aspects that can delay proper phage treatment is that phages are not always freely available for testing in patients. The characteristics of thousands of phages, including electron microscopy and genome, are known and reported in the literature; however, the existing phage collections (e.g., the Félix d’Hérelle Reference Center for bacterial viruses of the Université of Laval and the American Type Culture Collection) have a limited number of phages. Meanwhile, the researchers isolating and characterizing new phages keep them at their collections, which are mostly only accessible through a Materials Transfer Agreement between the supplier of the phage and the recipient, who have to follow the terms of the signed agreement.

Also, phage manufacturing procedures should be subject to a discussion to harmonize the methodologies needed to produce viable, stable, and endotoxin-free phage formulations, using GMP conditions if necessary; however, this last condition is associated with a high financial challenge. A recent revision of the regulation 536/2014 (Directive 2001/20/EC) provides some flexibility or even exemption from GMP production when trials occur in the same hospital, health center, or clinic authorized in an EC Member State.

Since the compassionate approach is patient-targeted, the formulation might have to be prepared locally at the hospital pharmacy and if not correctly handled, can increase the risk of product cross-contamination by the pharmacist.

The specificity of phages is one of the significant issues that can arise and compromise treatment. Phage collections provide a small vial of the phage along with the host requiring further production, purification, and evaluation against the bacterial isolate. These collections sell these phages at different price ranges and once these phages are tested in the clinical isolate of interest, their specificity issues might even render them ineffective for a patient. Another main drawback of the application of phage therapy to patients is that a phage purchased from a collection can be utterly inefficient towards the clinical isolate that is causing an infection. A collection of hundreds of phages should exist in healthcare providing systems or a free- or small-fee-access approach to allow an initial susceptibility test to rule out the treatment with a non-infective phage. According to Pirnay et al. (2018), similarly to the pharmaceutic industry, researchers should be monetarily rewarded for their work in isolating, characterizing, and optimizing the phages to compensate them for their efforts [188] as well as the research institutions involved.

## 10. Conclusions

Non-healing wounds are an increasing health and global financial burden and are challenging to treat due to biofilm-forming and MDR bacteria. Phage therapy has received renewed interest due mainly to a vast number of successful published results obtained from patient cases that had undergone traditional antibiotherapy but without success. Phages have proved to have an active bactericidal activity, even against antibiotic-resistant bacteria, and are a promising alternative to conventional antibiotics. Phage formulations have evolved from phages in liquid solutions (e.g., filtered phage lysate solutions in saline containing preservative agents such as 8-hydroxyquinoline sulfate monohydrate or chinazolin) to different delivery systems that have proved successful in different laboratory models. However, phage therapy in patients in need must follow the proper regulatory framework.

This would ensure treatment with reliable phage delivery at the site of infection and that the stability would remain consistent throughout the treatment duration. Although the recent declarations and decisions have opened paths to a more accessible legal use of phage therapy under temporary use authorizations as new drugs, discussions should continue until phage therapy is made legal as a standard medical practice.

## Figures and Tables

**Table 1:** Different phage therapy approaches reported using *in vitro*, *ex vivo*, and *in vivo* animal and human models of chronic wounds.

Authors	Year	Individual/Cocktail Phages	Phage Name	Host Organism										Study Model				Dosage	Main Conclusions
				*S. aureus*	*E. coli*	*P. aeruginosa*	*A. baumannii*	*P. mirabilis*	*K. pneumoniae*	*E. faecium*	*E. faecalis*	*Streptococcus* sp.	*Proteus* sp.	*In vitro*	*In vivo*	*Ex vivo*	Human		
Non-encapsulated phage strategies																			
[125]	2013	Cocktail of 5 species-specific phages	F44/10	×											×			10^8^ to 10^9^ PFU/mL	Decrease of the bacterial counts. Wound healing improvement.
			F125/10	×															
			F510/08			×													
			F770/05			×													
			F1245/05				×												
[126]	2013	Species-specific phage	^1^ND	×											×			10^6^ PFU/mL	The combination of phage treatment with debridement improved healing and reduced bacterial counts.
[161]	2014	Cocktail of 3 species-specific phages	14/1			×											×	10^9^ PFU/mL	The topical application of this cocktail did not show any adverse effects; however, its efficacy was not adequately studied.
			PNM			×													
			ISP	×															
[162]	2017	Individual phage and cocktails	Sb-1	×													×	10^7^ PFU/mL	No allergic reactions observed and after seven days, bacterial loads decreased and wounds improved.
			Pyophage	×	×	×						×	×						
			Fersis	×								×							
[122]	2018	Cocktail of 2 species-specific phages	vB_EcoS_CEB_EC3a		×									×		×		10^9^ PFU/mL	Phage-honey acted synergistically and reduced CFU counts.
			vB_PaeP_PAO1-D			×													
[123]	2018	Individual phage and cocktails	DRA88	×												×		4 h treatment: 5 μL containing 10^6^ PFU/mL 24 h treatment: 10^7^ PFU/mL	Reduction of viable cells and biofilm formation.
			SAB4328-A	×															
[134]	2019	Cocktail of 3 species-specific phages	ND	×	×	×											×	0.1 mL/cm^2^ and 10^9^ PFU/mL	3 to 5 doses of topical phage resulted in no signs of infection. Seven patients achieved complete healing on day 21.
[121]	2019	Individual phage and cocktails	EC7a		×									×		×		100 µL of phage or phage cocktail at different multiplicities of infection (MOI)	Decrease of viable cells in biofilms formed on porcine skins for phages applied alone or in a cocktail.
			EC7b		×														
			EC3a		×														
			P2				×												
			P1				×												
			AB7a				×												
			PA1			×													
			PA4			×													
			Pm5460					×											
			Pm5461					×											
[163]	2019	Individual phage and cocktails	vB_EfaS-Zip							×				×				10^8^ PFU/mL	Three hours of treatment with the phage cocktail led to a 2.5 log CFU/mL reduction.
			vB_EfaP-Max								×								
Injection of phages into the soft tissue																			
[164]	2017	Cocktail of 2 species-specific phages	BFC1			×											×	ND	Blood cultures were negative for the presence of bacteria. However, the wounds remained colonized. The patient succumbed to blood sepsis derived from *Klebsiella pneumoniae* colonization.
[131]	2018	Species-specific phage	Sb-1	×													×	PFU/mL not referred. Injections of 0.7 cc of phage, once a week for seven weeks (total of 4.9 cc)	Ulcer healed. Re-ossification of the distal phalanx occurred and the patient discharged after three months.
Phage encapsulation in liposomes																			
[165]	2017	Cocktail	KØ1						×						×			10^8^ PFU/mL	Phage cocktail entrapped within liposomes reduced more cells and led to a faster resolution of the infection.
			KØ2						×										
			KØ3						×										
			KØ4						×										
			KØ5						×										
[166]	2018	Cocktail	MR-5	×										×	×			10^9^ PFU/50 µL	Cocktail of two phages reduced more bacteria and led to faster healings compared to individual phages. Liposomal phage cocktail entrapment persisted longer at the wound site.
			MR-10	×															
Phage encapsulation in nanospheres																			
[167]	2015	Species-specific phage	K	×										×				10^9^ PFU/mL	The formulation of the phage with poly(N-isopropylacrylamid) nanospheres copolymerized with allylamine, anchored onto a simulated dressing via plasma deposition, demonstrated to lyse bacterial isolates under body temperature of 37 °C.
Incorporation of bacteriophage into emulsions																			
[168]	2014	Species-specific phage	K	×										×				10^5^ PFU/mL	Higher antibacterial activity found in phage-loaded emulsions compared to free phages. The three strains studied here were rapidly and entirely killed by nanoemulsions.
Incorporation of phages within adhesives																			
[169]	2019	Species-specific phage	PA5			×								×				10^11^ PFU	Phage immobilization within fibrin glue resulted in the release of high titers of viable phages during 11 days.
Incorporation of phages within fibers																			
[170]	2017	Species-specific phage	vB_Pae_Kakheti25			×								×				ND	The use of polycaprolactone to immobilize the phage eradicated the bacterium.
[171]	2018	Species-specific phage	T4		×									×	×			10^13^ PFU/mL	Polycaprolactone/collagen I B *in vivo* fully degraded in 8 weeks without adverse reactions to muscle and subcutaneous tissues.
Incorporation of phages within hydrogels																			
[172]	2014	Species-specific phage	ΦK	×										×				10^8^ PFU/mL	Phage release facilitated by hyaluronidase, which degraded the hyaluronic acid methacrylate present in the upper layer of the hydrogel, promoting the subsequent killing of bacteria.
[173]	2019	Species-specific phage	MR10	×										×	×			MOI 10	PVA-Sodium alginate hydrogel-based dressings with minocycline and phages were effective against infected burn wounds, reducing bacterial colonization and inflammation significantly.
			Kpn5						×										
			PA5			×													

1 Not defined.

## References

[1] Lazarus G.S., Cooper D.M., Knighton D.R., Margolis D.J., Percoraro R.E., Rodeheaver G., Robson M.C. (1994). Definitions and guidelines for assessment of wounds and evaluation of healing. Wound Repair Regen..

[2] Siddiqui A.R., Bernstein J.M. (2010). Chronic wound infection: Facts and controversies. Clin. Dermatol..

[3] Hu M.S., Borrelli M.R., Lorenz H.P., Longaker M.T., Wan D.C. (2018). Mesenchymal stromal cells and cutaneous wound healing: A comprehensive review of the background, role, and therapeutic potential. Stem Cells Int..

[4] Aljghami M.E., Saboor S., Amini-Nik S. (2019). Emerging innovative wound dressings. Ann. Biomed. Eng..

[5] Mustoe T.A., O’Shaughnessy K., Kloeters O. (2006). Chronic wound pathogenesis and current treatment strategies: A unifying hypothesis. Plast. Reconstr. Surg..

[6] Kim H.S., Sun X., Lee J.-H., Kim H.-W., Fu X., Leong K.W. (2018). Advanced drug delivery systems and artificial skin grafts for skin wound healing. Adv. Drug Deliv. Rev..

[7] Chang F., Yang C.W., Lu W. (2007). Chronic wound: Pathogenesis and current treatments. Acad. J. Second Mil. Med. Univ..

[8] Macdonald J. (2009). Global Initiative for Wound and Lymphoedema Care (GIWLC). J. Lymphoedema.

[9] Reilly E., Karakousis G., Schrag S., Stawicki S. (2007). Pressure ulcers in the intensive care unit: The ‘forgotten’enemy. Opus.

[10] World Health Organization Antimicrobial Resistance. https://www.who.int/news-room/fact-sheets/detail/antimicrobial-resistance.

[11] Manohar P., Nachimuthu R., Lopes B.S. (2018). The therapeutic potential of bacteriophages targeting gram-negative bacteria using *Galleria mellonella* infection model. BMC Microbiol..

[12] Pence B.D., Woods J.A. (2014). Exercise, obesity, and cutaneous wound healing: Evidence from rodent and human studies. Adv. Wound Care.

[13] Werdin F., Tennenhaus M., Schaller H. (2009). Evidence-based management strategies for treatment of chronic wounds. ePlasty Open Access J. Plast. Surg..

[14] Cazander G., Pritchard D.I., Nigam Y., Jung W., Nibbering P.H. (2013). Multiple actions of *Lucilia sericata* larvae in hard-to-heal wounds: Larval secretions contain molecules that accelerate wound healing, reduce chronic inflammation and inhibit bacterial infection. BioEssays.

[15] Graves N., Zheng H. (2014). The prevalence and incidence of chronic wounds: A literature review. Wound Pract. Res. J. Aust. Wound Manag. Assoc..

[16] Martinengo L., Olsson M., Bajpai R., Soljak M., Upton Z., Schmidtchen A., Car J., Järbrink K. (2018). Prevalence of chronic wounds in the general population: Systematic review and meta-analysis of observational studies. Ann. Epidemiol..

[17] Ellis S., Lin E.J., Tartar D. (2018). Immunology of wound healing. Curr. Dermatol. Rep..

[18] Kalan L.R., Brennan M.B. (2019). The role of the microbiome in nonhealing diabetic wounds. Ann. N. Y. Acad. Sci..

[19] Krasner D., Rodeheaver G., Sibbald R. (2007). Chronic Wound Care: A Clinical Source Book for Healthcare Professionals.

[20] Vos T., Allen C., Arora M., Barber R.M., Brown A., Carter A., Casey D.C., Charlson F.J., Chen A.Z., Coggeshall M. (2016). Global, regional, and national incidence, prevalence, and years lived with disability for 310 diseases and injuries, 1990–2015: A systematic analysis for the Global Burden of Disease Study 2015. Lancet.

[21] James S.L., Abate D., Abate K.H., Abay S.M., Abbafati C., Abbasi N., Abbastabar H., Abd-Allah F., Abdela J., Abdelalim A. (2018). Global, regional, and national incidence, prevalence, and years lived with disability for 354 diseases and injuries for 195 countries and territories, 1990–2017: A systematic analysis for the Global Burden of Disease Study 2017. Lancet.

[22] Lim H.W., Collins S.A.B., Resneck J.S., Bolognia J.L., Hodge J.A., Rohrer T.A., Van Beek M.J., Margolis D.J., Sober A.J., Weinstock M.A. (2017). The burden of skin disease in the United States. J. Am. Acad. Dermatol..

[23] Sen C.K., Gordillo G.M., Roy S., Kirsner R., Lambert L., Hunt T.K., Gottrup F., Gurtner G.C., Longaker M.T. (2009). Human skin wounds: A major and snowballing threat to public health and the economy: Perspective article. Wound Repair Regen..

[24] Dealey C., Posnett J., Walker A. (2012). The cost of pressure ulcers in the United Kingdom. J. Wound Care.

[25] Nussbaum S.R., Carter M.J., Fife C.E., DaVanzo J., Haught R., Nusgart M., Cartwright D. (2018). An economic evaluation of the impact, cost, and Medicare policy implications of chronic nonhealing wounds. Value Health.

[26] Wang P.-H., Huang B.-S., Horng H.-C., Yeh C.-C., Chen Y.-J. (2018). Wound healing. J. Chin. Med. Assoc..

[27] Roşca A.-M., Ţuţuianu R., Domnica Titorencu I. (2018). Mesenchymal stromal cells derived exosomes as tools for chronic wound healing therapy. Rom. J. Morphol. Embryol..

[28] Ibrahim N.‘I., Wong S.K., Mohamed I.N., Mohamed N., Chin K.-Y., Ima-Nirwana S., Shuid A.N. (2018). Wound healing properties of selected natural products. Int. J. Environ. Res. Public Health.

[29] Powers J.G., Higham C., Broussard K., Phillips T.J. (2016). Wound healing and treating wounds: Chronic wound care and management. J. Am. Acad. Dermatol..

[30] Swanson T., Angel D., Sussman G., Cooper R., Haesler E., Ousey K., Carville K., Fletcher J., Kalan L., Keast D. (2016). International Wound Infection Institute (IWII) Wound infection in clinical practice: Principles of best practice. Wounds Int..

[31] Bowler P.G., Duerden B.I., Armstrong D.G. (2001). Wound microbiology and associated approaches to wound management. Clin. Microbiol. Rev..

[32] Méric G., Mageiros L., Pensar J., Laabei M., Yahara K., Pascoe B., Kittiwan N., Tadee P., Post V., Lamble S. (2018). Disease-associated genotypes of the commensal skin bacterium *Staphylococcus Epidermidis*. Nat. Commun..

[33] Evangelista S., Guimaraes N., Garcia N., Santos S., Oliveira A. (2019). Effectiveness of manual versus automated cleaning on *Staphylococcus epidermidis* biofilm removal from the surface of surgical instruments. Am. J. Infect. Control.

[34] Mody L., Washer L.L., Kaye K.S., Gibson K., Saint S., Reyes K., Cassone M., Mantey J., Cao J., Altamimi S. (2019). Multidrug-resistant organisms in hospitals: What is on patient hands and in their rooms?. Clin. Infect. Dis..

[35] Carikas B.K., Matthews S. (2019). Hospital privacy curtains – What’s hanging around?. Dissector.

[36] Moremi N., Claus H., Silago V., Kabage P., Abednego R., Matee M., Vogel U., Mshana S.E. (2019). Hospital surface contamination with antimicrobial-resistant Gram-negative organisms in Tanzanian regional and tertiary hospitals: The need to improve environmental cleaning. J. Hosp. Infect..

[37] Haque M., Sartelli M., McKimm J., Bakar M.A. (2018). Health care-associated infections—An overview. Infect. Drug Resist..

[38] Patel S. (2007). Understanding wound infection and colonisation. Wound Essentials.

[39] Gjødsbøl K., Christensen J.J., Karlsmark T., Jørgensen B., Klein B.M., Krogfelt K.A. (2006). Multiple bacterial species reside in chronic wounds: A longitudinal study. Int. Wound J..

[40] Oliveira A., Ribeiro H.G., Silva A.C., Silva M.D., Sousa J.C., Rodrigues C.F., Melo L.D.R., Henriques A.F., Sillankorva S. (2017). Synergistic antimicrobial interaction between honey and phage against *Escherichia coli* biofilms. Front. Microbiol..

[41] Jneid J., Cassir N., Schuldiner S., Jourdan N., Sotto A., Lavigne J.P., Scola B. (2018). La Exploring the microbiota of diabetic foot infections with culturomics. Front. Cell. Infect. Microbiol..

[42] Otta S., Debata N.K., Swain B. (2019). Bacteriological profile of diabetic foot ulcers. J. Health Res..

[43] Ogba O.M., Nsan E., Eyam E.S. (2019). Aerobic bacteria associated with diabetic foot ulcers and their susceptibility pattern. Biomed. Dermatol..

[44] Lachiewicz A.M., Hauck C.G., Weber D.J., Cairns B.A., Van Duin D. (2017). Bacterial Infections after Burn Injuries: Impact of Multidrug Resistance. Clin. Infect. Dis..

[45] Oruko R.O., Odiyo J.O., Edokpayi J.N. (2019). The role of leather microbes in human health. IntechOpen.

[46] Findley K., Oh J., Yang J., Conlan S., Deming C., Meyer J.A., Schoenfeld D., Nomicos E., Park M., Becker J. (2013). Topographic diversity of fungal and bacterial communities in human skin. Nature.

[47] Cui L., Morris A., Ghedin E. (2013). The human mycobiome in health and disease. Genome Med..

[48] Hansson C., Hoborn J., Möller A., Swanbeck G. (1995). The microbial flora in venous leg ulcers without clinical signs of infection. Repeated culture using a validated standardised microbiological technique. Acta Derm. Venereol..

[49] Krüger W., Vielreicher S., Kapitan M., Jacobsen I.D., Niemiec M.J. (2019). Fungal-bacterial interactions: In health and disease. Candida albicans Cell. Mol. Biol. Second Ed..

[50] Kalan L., Loesche M., Hodkinson B.P., Heilmann K., Ruthel G., Gardner S.E., Grice E.A. (2016). Redefining the chronic-wound microbiome: Fungal communities are prevalent, dynamic, and associated with delayed healing. MBio.

[51] Chellan G., Shivaprakash S., Ramaiyar S.K., Varma A.K., Varma N., Sukumaran M.T., Vasukutty J.R., Bal A., Kumar H. (2010). Spectrum and prevalence of fungi infecting deep tissues of lower-limb wounds in patients with type 2 diabetes. J. Clin. Microbiol..

[52] Gupta N., Haque A., Mukhopadhyay G., Narayan R.P., Prasad R. (2005). Interactions between bacteria and *Candida* in the burn wound. Burns.

[53] Kalan L., Grice E.A. (2018). Fungi in the wound microbiome. Adv. Wound Care.

[54] Dowd S.E., Delton Hanson J., Rees E., Wolcott R.D., Zischau A.M., Sun Y., White J., Smith D.M., Kennedy J., Jones C.E. (2011). Survey of fungi and yeast in polymicrobial infections in chronic wounds. J. Wound Care.

[55] Que A.T., Nguyen N.M.T., Do N.A., Nguyen N.L., Tran N.D., Le T.A. (2019). Infection of burn wound by *Aspergillus fumigatus* with gross appearance of fungal colonies. Med. Mycol. Case Rep..

[56] Costerton J.W., Geesey G.G., Cheng K.J. (1978). How bacteria stick in. Sci. Am. Inc.

[57] Percival S.L., Hill K.E., Williams D.W., Hooper S.J., Thomas D.W., Costerton J.W. (2012). A review of the scientific evidence for biofilms in wounds. Wound Repair Regen..

[58] Clinton A., Carter T. (2015). Chronic wound biofilms: Pathogenesis and potential therapies. Lab. Med..

[59] Liu J., Fu K., Wu C., Qin K., Li F., Zhou L. (2018). “In-group” communication in marine *Vibrio*: A review of N-acyl homoserine lactones-driven quorum sensing. Front. Cell. Infect. Microbiol..

[60] Maunders E., Welch M. (2017). Matrix exopolysaccharides; the sticky side of biofilm formation. FEMS Microbiol. Lett..

[61] Costerton J.W., Lewandowski Z., Caldwell D.E., Korber D.R., Lappin-Scott H.M. (1995). Microbial biofilms. Annu. Rev. Microbiol..

[62] Klausen M., Heydorn A., Ragas P., Lambertsen L., Aaes-Jørgensen A., Molin S., Tolker-Nielsen T. (2003). Biofilm formation by *Pseudomonas aeruginosa* wild type, flagella and type IV pili mutants. Mol. Microbiol..

[63] Stewart P.S. (2003). Diffusion in biofilms. J. Bacteriol..

[64] Donlan R.M. (2002). Biofilms: Microbial life on surfaces. Emerg. Infect. Dis..

[65] Pires D.P., Melo L.D.R., Vilas Boas D., Sillankorva S., Azeredo J. (2017). Phage therapy as an alternative or complementary strategy to prevent and control biofilm-related infections. Curr. Opin. Microbiol..

[66] Shah D., Zhang Z., Khodursky A., Kaldalu N., Kurg K., Lewis K. (2006). Persisters: A distinct physiological state of *E. coli*. BMC Microbiol..

[67] Kwan B.W., Valenta J.A., Benedik M.J., Wood T.K. (2013). Arrested protein synthesis increases persister-like cell formation. Antimicrob. Agents Chemother..

[68] Wood T.K., Knabel S.J., Kwan B.W. (2013). Bacterial persister cell formation and dormancy. Appl. Environ. Microbiol..

[69] Conlon B.P., Rowe S.E., Gandt A.B., Nuxoll A.S., Donegan N.P., Zalis E.A., Clair G., Adkins J.N., Cheung A.L., Lewis K. (2016). Persister formation in *Staphylococcus aureus* is associated with ATP depletion. Nat. Microbiol..

[70] Lewis K. (2010). Persister Cells. Annu. Rev. Microbiol..

[71] Ng W.-L., Bassler B.L. (2009). Bacterial quorum-sensing network architectures. Annu. Rev. Genet..

[72] Eickhoff M.J., Bassler B.L. (2018). SnapShot: Bacterial Quorum Sensing. Cell.

[73] Mendoza R.A., Hsieh J.C., Galiano R.D. (2019). The impact of biofilm formation on wound healing. IntechOpen.

[74] Leid J.G. (2009). Bacterial biofilms resist key host defenses. Microbe.

[75] Tacconelli E., Margrini N. Global Priority List of Antibiotic-Resistant Bacteria to Guide Research, Discovery, and Development of New Antibiotics. https://www.who.int/medicines/publications/WHO-PPL-Short_Summary_25Feb-ET_NM_WHO.pdf?ua=1.

[76] Gaddy J.A., Actis L.A. (2009). Regulation of *Acinetobacter baumannii* biofilm formation. Future Microbiol..

[77] Rasamiravaka T., Labtani Q., Duez P., El Jaziri M. (2015). The formation of biofilms by *Pseudomonas aeruginosa*: A review of the natural and synthetic compounds interfering with control mechanisms. Biomed Res. Int..

[78] Vuotto C., Longo F., Balice M.P., Donelli G., Varaldo P.E. (2014). Antibiotic resistance related to biofilm formation in *Klebsiella pneumoniae*. Pathogens.

[79] Beloin C., Roux A., Ghigo J.M. (2008). Escherichia coli biofilms. Current Topics in Microbiology and Immunology-Bacterial Biofilms.

[80] Bianchi T., Wolcott R.D., Peghetti A., Leaper D., Cutting K., Polignano R., Rosa Rita Z., Moscatelli A., Greco A., Romanelli M. (2016). Recommendations for the management of biofilm: A consensus document. J. Wound Care.

[81] Parsek M.R., Singh P.K. (2003). Bacterial biofilms: An emerging link to disease pathogenesis. Annu. Rev. Microbiol..

[82] Vachhrajani V., Khakhkhar P. (2020). Other types of dressings. Science of Wound Healing and Dressing Materials.

[83] Molan P.C. (1992). The antibacterial activity of honey: 1. The nature of the antibacterial activity. Bee World.

[84] Brudzynski K. (2006). Effect of hydrogen peroxide on antibacterial activities of Canadian honeys. Can. J. Microbiol..

[85] Gethin G.T., Cowman S., Conroy R.M. (2008). The impact of Manuka honey dressings on the surface pH of chronic wounds. Int. Wound J..

[86] Majtan J., Bohova J., Prochazka E., Klaudiny J. (2014). Methylglyoxal may affect hydrogen peroxide accumulation in manuka honey through the inhibition of glucose oxidase. J. Med. Food.

[87] Dhivya S., Padma V.V., Santhini E. (2015). Wound dressings-A review. BioMedicine.

[88] Negut I., Grumezescu V., Grumezescu A.M. (2018). Treatment strategies for infected wounds. Molecules.

[89] Mostafalu P., Kiaee G., Giatsidis G., Khalilpour A., Nabavinia M., Dokmeci M.R., Sonkusale S., Orgill D.P., Tamayol A., Khademhosseini A. (2017). A textile dressing for temporal and dosage controlled drug delivery. Adv. Funct. Mater..

[90] Schultz G.S., Sibbald R.G., Falanga V., Ayello E.A., Dowsett C., Harding K., Romanelli M., Stacey M.C. (2003). Wound bed preparation: A systematic approach to wound management. Wound Repair Regen..

[91] Jull A.B., Arroll B., Parag V., Waters J. (2012). Pentoxifylline for treating venous leg ulcers. Cochrane Database Syst. Rev..

[92] Cruciani M., Lipsky B.A., Mengoli C., de Lalla F. (2013). Granulocyte-colony stimulating factors as adjunctive therapy for diabetic foot infections. Cochrane Database Syst. Rev..

[93] Kranke P., Bennett M.H., Martyn-St James M., Schnabel A., Debus S.E., Weibel S. (2015). Hyperbaric oxygen therapy for chronic wounds. Cochrane Database Syst. Rev..

[94] Helen T., Liz C., Laura C., Illary S., Martin B., Hannah B., Ian C., Jo D., Chris F., Rachael F. (2019). Aspirin versus placebo for the treatment of venous leg ulcers-A phase II, pilot, randomised trial (AVURT). Trials.

[95] Polera N., Badolato M., Perri F., Carullo G., Aiello F. (2018). Quercetin and its natural sources in wound healing management. Curr. Med. Chem..

[96] Wu B., Lu J., Yang M., Xu T. (2016). Sulodexide for treating venous leg ulcers. Cochrane Database Syst. Rev..

[97] Jeschke M.G., Kamolz L.P., Sjöberg F., Wolf S.E., Jeschke M.G., Kamolz L.P., Sjöberg F., Wolf S.E. (2012). Handbook of Burns: Acute Burn Care, Volume 1.

[98] Murray C.K. (2017). Field wound care: Prophylactic antibiotics. Wilderness Environ. Med..

[99] Bigliardi P.L., Alsagoff S.A.L., El-Kafrawi H.Y., Pyon J.K., Wa C.T.C., Villa M.A. (2017). Povidone iodine in wound healing: A review of current concepts and practices. Int. J. Surg..

[100] Kramer A., Dissemond J., Kim S., Willy C., Mayer D., Papke R., Tuchmann F., Assadian O. (2018). Consensus on wound antisepsis: Update 2018. Skin Pharmacol. Physiol..

[101] Schedler K., Assadian O., Brautferger U., Müller G., Koburger T., Classen S., Kramer A. (2017). Proposed phase 2/ step 2 *in-vitro* test on basis of EN 14561 for standardised testing of the wound antiseptics PVP-iodine, chlorhexidine digluconate, polihexanide and octenidine dihydrochloride. BMC Infect. Dis..

[102] Leaper D.J., Schultz G., Carville K., Fletcher J., Swanson T., Drake R. (2012). Extending the TIME concept: What have we learned in the past 10 years?. Int. Wound J..

[103] König B., Reimer K., Fleischer W., König W. (1997). Effects of Betaisodona^®^ on parameters of host defense. Dermatology.

[104] Costa S.S., Viveiros M., Amaral L., Couto I. (2013). Multidrug efflux pumps in *Staphylococcus aureus*: An update. Open Microbiol. J..

[105] Poole K. (2007). Efflux pumps as antimicrobial resistance mechanisms. Ann. Med..

[106] Park S.R., Kim J.W., Jun H.S., Roh J.Y., Lee H.Y., Hong I.S. (2018). Stem cell secretome and its effect on cellular mechanisms relevant to wound healing. Mol. Ther..

[107] Kutter E., Kuhl S., Abedon S., Alavidze Z., Gvasalia G., De Vos D., Gogokhia L. (2010). Phage therapy in clinical practice: Treatment of human infections. Curr. Pharm. Biotechnol..

[108] Maciejewska B., Olszak T., Drulis-Kawa Z. (2018). Applications of bacteriophages versus phage enzymes to combat and cure bacterial infections: An ambitious and also a realistic application?. Appl. Microbiol. Biotechnol..

[109] Dy R.L., Rigano L.A., Fineran P.C. (2018). Phage-based biocontrol strategies and their application in agriculture and aquaculture. Biochem. Soc. Trans..

[110] Chanishvili N. (2012). Phage therapy-History from Twort and d’Herelle through Soviet experience to current approaches. Advances in Virus Research.

[111] D’Herelle F. (1931). Bacteriophage as a treatment in acute medical and surgical infections. Bull. N. Y. Acad. Med..

[112] Abedon S.T., Kuhl S.J., Blasdel B.G., Kutter E.M. (2011). Phage treatment of human infections. Bacteriophage.

[113] Sulakvelidze A., Alavidze Z., Morris G.J. (2001). Bacteriophage therapy. Antimicrob Agents Chemother.

[114] Summers W.C. (1999). Felix d’Hérelle and the Origins of Molecular Biology.

[115] Bruynoghe R., Maisin J. (1921). Essais de the rapeutique au moyen du bacteriophage. C. R. Soc. Biol..

[116] Myelnikov D. (2018). An alternative cure: The adoption and survival of bacteriophage therapy in the USSR, 1922-1955. J. Hist. Med. Allied Sci..

[117] Malik D.J., Sokolov I.J., Vinner G.K., Mancuso F., Cinquerrui S., Vladisavljevic G.T., Clokie M.R.J., Garton N.J., Stapley A.G.F., Kirpichnikova A. (2017). Formulation, stabilisation and encapsulation of bacteriophage for phage therapy. Adv. Colloid Interface Sci..

[118] Sarker S.A., Sultana S., Reuteler G., Moine D., Descombes P., Charton F., Bourdin G., McCallin S., Ngom-Bru C., Neville T. (2016). Oral phage therapy of acute bacterial diarrhea with two coliphage preparations: A randomized trial in children from Bangladesh. EBioMedicine.

[119] Qadir M.I., Mobeen T., Masood A. (2018). Phage therapy: Progress in pharmacokinetics. Brazilian J. Pharm. Sci..

[120] McCallin S., Sarker S.A., Sultana S., Oechslin F., Brüssow H. (2018). Metagenome analysis of Russian and Georgian Pyophage cocktails and a placebo-controlled safety trial of single phage versus phage cocktail in healthy *Staphylococcus aureus* carriers. Environ. Microbiol..

[121] Milho C., Andrade M., Vilas Boas D., Alves D., Sillankorva S. (2019). Antimicrobial assessment of phage therapy using a porcine model of biofilm infection. Int. J. Pharm..

[122] Oliveira A., Sousa J.C., Silva A.C., Melo L.D.R., Sillankorva S. (2018). Chestnut honey and bacteriophage application to control *Pseudomonas aeruginosa* and *Escherichia coli* biofilms: Evaluation in an *ex vivo* wound model. Front. Microbiol..

[123] Alves D.R., Booth S.P., Scavone P., Schellenberger P., Salvage J., Dedi C., Thet N.-T., Jenkins A.T.A., Waters R., Ng K.W. (2018). Development of a high-throughput *ex-vivo* burn wound model using porcine skin, and its application to evaluate new approaches to control wound infection. Front. Cell. Infect. Microbiol..

[124] Gomes-Fernandes M., Laabei M., Pagan N., Hidalgo J., Molinos S., Villar Hernandez R., Domínguez-Villanueva D., Jenkins A.T.A., Lacoma A., Prat C. (2017). Accessory gene regulator (Agr) functionality in *Staphylococcus aureus* derived from lower respiratory tract infections. PLoS ONE.

[125] Mendes J.J., Leandro C., Corte-Real S., Barbosa R., Cavaco-Silva P., Melo-Cristino J., Gõrski A., Garcia M. (2013). Wound healing potential of topical bacteriophage therapy on diabetic cutaneous wounds. Wound Repair Regen..

[126] Seth A.K., Geringer M.R., Nguyen K.T., Agnew S.P., Dumanian Z., Galiano R.D., Leung K.P., Mustoe T.A., Hong S.J. (2013). Bacteriophage therapy for *Staphylococcus aureus* biofilm-infected wounds: A new approach to chronic wound care. Plast. Reconstr. Surg..

[127] Djebara S., Maussen C., De Vos D., Merabishvili M., Damanet B., Pang K.W., De Leenheer P., Strachinaru I., Soentjens P., Pirnay J.P. (2019). Processing phage therapy requests in a Brussels military hospital: Lessons identified. Viruses.

[128] Jault P., Leclerc T., Jennes S., Pirnay J.P., Que Y.A., Resch G., Rousseau A.F., Ravat F., Carsin H., Le Floch R. (2019). Efficacy and tolerability of a cocktail of bacteriophages to treat burn wounds infected by *Pseudomonas aeruginosa* (PhagoBurn): A randomised, controlled, double-blind phase 1/2 trial. Lancet Infect. Dis..

[129] Rhoads D.D., Wolcott R.D., Kuskowski M.A., Wolcott B.M., Ward L.S., Sulakvelidze A. (2009). Bacteriophage therapy of venous leg ulcers in humans: Results of a phase I safety trial. J. Wound Care.

[130] Fish R., Kutter E., Wheat G., Blasdel B., Kutateladze M., Kuhl S. (2016). Bacteriophage treatment of intransigent diabetic toe ulcers: A case series. J. Wound Care.

[131] Fish R., Kutter E., Wheat G., Blasdel B., Kutateladze M., Kuhl S. (2018). Compassionate use of bacteriophage therapy for foot ulcer treatment as an effective step for moving toward clinical trials. Methods in Molecular Biology.

[132] Morozova V.V., Kozlova Y.N., Ganichev D.A., Tikunova N.V. (2018). Bacteriophage treatment of infected diabetic foot ulcers. Methods in Molecular Biology.

[133] Abo-elmaaty S., El Dougdoug N.K., Hazaa M.M. (2016). Improved antibacterial efficacy of bacteriophage-cosmetic formulation for treatment of *Staphylococcus aureus in vitro*. Ann. Agric. Sci..

[134] Gupta P., Singh H.S., Shukla V.K., Nath G., Bhartiya S.K. (2019). Bacteriophage therapy of chronic nonhealing wound: Clinical study. Int. J. Low. Extrem. Wounds.

[135] Ooi M.L., Drilling A.J., Morales S., Fong S., Moraitis S., MacIas-Valle L., Vreugde S., Psaltis A.J., Wormald P.J. (2019). Safety and tolerability of bacteriophage therapy for chronic rhinosinusitis due to *Staphylococcus aureus*. JAMA Otolaryngol.-Head Neck Surg..

[136] Tagliaferri T.L., Jansen M., Horz H.P. (2019). Fighting pathogenic bacteria on two fronts: Phages and antibiotics as combined strategy. Front. Cell. Infect. Microbiol..

[137] Furfaro L.L., Payne M.S., Chang B.J. (2018). Bacteriophage therapy: Clinical trials and regulatory hurdles. Front. Cell. Infect. Microbiol..

[138] Kortright K.E., Chan B.K., Koff J.L., Turner P.E. (2019). Phage therapy: A renewed approach to combat antibiotic-resistant bacteria. Cell Host Microbe.

[139] Górski A., Miedzybrodzki R., Weber-Dabrowska B., Fortuna W., Letkiewicz S., Rogóz P., Jończyk-Matysiak E., Dabrowska K., Majewska J., Borysowski J. (2016). Phage therapy: Combating infections with potential for evolving from merely a treatment for complications to targeting diseases. Front. Microbiol..

[140] Huh H., Wong S., St. Jean J., Slavcev R. (2019). Bacteriophage interactions with mammalian tissue: Therapeutic applications. Adv. Drug Deliv. Rev..

[141] Fauconnier A. (2019). Phage therapy regulation: From night to dawn. Viruses.

[142] Weld R.J., Butts C., Heinemann J.A. (2004). Models of phage growth and their applicability to phage therapy. J. Theor. Biol..

[143] Weiss M., Denou E., Bruttin A., Serra-Moreno R., Dillmann M.L., Brüssow H. (2009). In vivo replication of T4 and T7 bacteriophages in germ-free mice colonized with *Escherichia coli*. Virology.

[144] Harada L.K., Silva E.C., Campos W.F., Del Fiol F.S., Vila M., Dąbrowska K., Krylov V.N., Balcão V.M. (2018). Biotechnological applications of bacteriophages: State of the art. Microbiol. Res..

[145] Chan B.K., Abedon S.T., Loc-Carrillo C. (2013). Phage cocktails and the future of phage therapy. Future Microbiol..

[146] Jasim H.N., Hafidh R.R., Abdulamir A.S. (2018). Formation of therapeutic phage cocktail and endolysin to highly studymulti-drug resistant *Acinetobacter baumannii*: *In vitro* and *in vivo*. Iran. J. Basic Med. Sci..

[147] Roach D.R., Debarbieux L. (2017). Phage therapy: Awakening a sleeping giant. Emerg. Top. Life Sci..

[148] Weber-Dabrowska B., Jończyk-Matysiak E., Zaczek M., Łobocka M., Łusiak-Szelachowska M., Górski A. (2016). Bacteriophage procurement for therapeutic purposes. Front. Microbiol..

[149] Schmerer M., Molineux I.J., Bull J.J. (2014). Synergy as a rationale for phage therapy using phage cocktails. PeerJ.

[150] Merabishvili M., Pirnay J.P., De Vos D. (2018). Guidelines to compose an ideal bacteriophage cocktail. Methods in Molecular Biology.

[151] Allen R.C., Pfrunder-Cardozo K.R., Meinel D., Egli A., Hall A.R. (2017). Associations among antibiotic and phage resistance phenotypes in natural and clinical *Escherichia coli* isolates. MBio.

[152] Ma Y., Pacan J.C., Wang Q., Xu Y., Huang X., Korenevsky A., Sabour P.M. (2008). Microencapsulation of bacteriophage felix o1 into chitosan-alginate microspheres for oral delivery. Appl. Environ. Microbiol..

[153] Colom J., Otero J., Cortés P., Llagostera M., Cano-Sarabia M., Maspoch D. (2015). Liposome-encapsulated bacteriophages for enhanced oral phage therapy against *Salmonella* spp.. Appl. Environ. Microbiol..

[154] McVay C.S., Velásquez M., Fralick J.A. (2007). Phage therapy of *Pseudomonas aeruginosa* infection in a mouse burn wound model. Antimicrob. Agents Chemother..

[155] Zhao J., Liu Y., Xiao C., He S., Yao H., Bao G. (2017). Efficacy of phage therapy in controlling rabbit colibacillosis and Changes in cecal microbiota. Front. Microbiol..

[156] Oliveira H., Sillankorva S., Merabishvili M., Kluskens L.D., Azeredo J. (2015). Unexploited opportunities for phage therapy. Front. Pharmacol..

[157] Brown T.L., Petrovski S., Chan H.T., Angove M.J., Tucci J. (2018). Semi-solid and solid dosage forms for the delivery of phage therapy to epithelia. Pharmaceuticals.

[158] El-Shibiny A., El-Sahhar S. (2017). Bacteriophages: The possible solution to treat infections caused by pathogenic bacteria. Can. J. Microbiol..

[159] Subils T., Aquili V., Ebner G., Balagué C. (2012). Effect of preservatives on Shiga toxigenic phages and Shiga toxin of *Escherichia coli* O157:H7. J. Food Prot..

[160] Merabishvili M., Monserez R., Van Belleghem J., Rose T., Jennes S., De Vos D., Verbeken G., Vaneechoutte M., Pirnay J.P. (2017). Stability of bacteriophages in burn wound care products. PLoS ONE.

[161] Rose T., Verbeken G., De Vos D., Merabishvili M., Vaneechoutte M., Lavigne R., Jennes S., Zizi M., Pirnay J.-P. (2014). Experimental phage therapy of burn wound infection: Difficult first steps. Int. J. Burns Trauma.

[162] Zhvania P., Hoyle N.S., Nadareishvili L., Nizharadze D., Kutateladze M. (2017). Phage therapy in a 16-year-old boy with netherton syndrome. Front. Med..

[163] Melo L.D.R., Ferreira R., Costa A.R., Oliveira H., Azeredo J. (2019). Efficacy and safety assessment of two enterococci phages in an *in vitro* biofilm wound model. Sci. Rep..

[164] Jennes S., Merabishvili M., Soentjens P., Pang K.W., Rose T., Keersebilck E., Soete O., François P.M., Teodorescu S., Verween G. (2017). Use of bacteriophages in the treatment of colistin-only-sensitive *Pseudomonas aeruginosa* septicaemia in a patient with acute kidney injury-a case report. Crit. Care.

[165] Chadha P., Katare O.P., Chhibber S. (2017). Liposome loaded phage cocktail: Enhanced therapeutic potential in resolving *Klebsiella pneumoniae* mediated burn wound infections. Burns.

[166] Chhibber S., Kaur J., Kaur S. (2018). Liposome entrapment of bacteriophages improves wound healing in a diabetic mouse MRSA infection. Front. Microbiol..

[167] Hathaway H., Alves D.R., Bean J., Esteban P.P., Ouadi K., Mark Sutton J., Jenkins A.T.A. (2015). Poly(N-isopropylacrylamide-co-allylamine) (PNIPAM-co-ALA) nanospheres for the thermally triggered release of bacteriophage K. Eur. J. Pharm. Biopharm..

[168] Esteban P.P., Alves D.R., Enright M.C., Bean J.E., Gaudion A., Jenkins A.T.A., Young A.E.R., Arnot T.C. (2014). Enhancement of the antimicrobial properties of bacteriophage-K via stabilization using oil-in-water nano-emulsions. Biotechnol. Prog..

[169] Rubalskii E., Ruemke S., Salmoukas C., Aleshkin A., Bochkareva S., Modin E., Mashaqi B., Boyle E.C., Boethig D., Rubalsky M. (2019). Fibrin glue as a local drug-delivery system for bacteriophage PA5. Sci. Rep..

[170] Nogueira F., Karumidze N., Kusradze I., Goderdzishvili M., Teixeira P., Gouveia I.C. (2017). Immobilization of bacteriophage in wound-dressing nanostructure. Nanomed. Nanotechnol. Biol. Med..

[171] Cheng W., Zhang Z., Xu R., Cai P., Kristensen P., Chen M., Huang Y. (2018). Incorporation of bacteriophages in polycaprolactone/collagen fibers for antibacterial hemostatic dual-function. J. Biomed. Mater. Res.-Part B Appl. Biomater..

[172] Bean J.E., Alves D.R., Laabei M., Esteban P.P., Thet N.T., Enright M.C., Jenkins A.T.A. (2014). Triggered release of bacteriophage K from agarose/hyaluronan hydrogel matrixes by *Staphylococcus aureus* virulence factors. Chem. Mater..

[173] Kaur P., Gondil V.S., Chhibber S. (2019). A novel wound dressing consisting of PVA-SA hybrid hydrogel membrane for topical delivery of bacteriophages and antibiotics. Int. J. Pharm..

[174] Choińska-Pulit A., Mituła P., Śliwka P., Łaba W., Skaradzińska A. (2015). Bacteriophage encapsulation: Trends and potential applications. Trends Food Sci. Technol..

[175] Cui H., Yuan L., Lin L. (2017). Novel chitosan film embedded with liposome-encapsulated phage for biocontrol of *Escherichia coli* O157:H7 in beef. Carbohydr. Polym..

[176] Ju Z., Sun W. (2017). Drug delivery vectors based on filamentous bacteriophages and phage-mimetic nanoparticles. Drug Deliv..

[177] Rägo L., Santoso B. (2008). Drug regulation: History, present and future. Drug Benefits and Risks: International Textbook of Clinical Pharmacology.

[178] Withington R. (2001). Regulatory issues for phage-based clinical products. J. Chem. Technol. Biotechnol..

[179] Verbeken G., De Vos D., Vaneechoutte M., Merabishvils M., Zizi M., Pirnay J.P. (2007). European regulatory conundrum of phage therapy. Future Microbiol..

[180] Cooper C.J., Mirzaei M.K., Nilsson A.S. (2016). Adapting drug approval pathways for bacteriophage-based therapeutics. Front. Microbiol..

[181] McCallin S., Sacher J.C., Zheng J., Chan B.K. (2019). Current state of compassionate phage therapy. Viruses.

[182] Kutateladze M., Adamia R. (2008). Phage therapy experience at the Eliava Institute. Med. Mal. Infect..

[183] (2013). World medical association declaration of helsinki: Ethical principles for medical research involving human subjects. JAMA-J. Am. Med. Assoc..

[184] Food and Drug Administration Expanded Access Program Report. https://www.fda.gov/media/119971/download.

[185] Paris V., Slawomirski L., Colbert A., Delaunay N., Oderkirk J. (2017). Innovation, access and value in pharmaceuticals. New Health Technologies—Managing Access, Value and Sustainability.

[186] Department of Health-Therapeutics Goods Administration Accessing Unapproved Products. https://www.tga.gov.au/accessing-unapproved-products.

[187] Department of Health-Therapeutics Goods Administration Personal Import Scheme. https://www.tga.gov.au/publication/personal-import-scheme.

[188] Pirnay J.P., Verbeken G., Ceyssens P.J., Huys I., de Vos D., Ameloot C., Fauconnier A. (2018). The magistral phage. Viruses.

[189] Fauconnier A. (2018). Guidelines for bacteriophage product certification. Methods Mol. Biol..

[190] Górski A., Międzybrodzki R., Łobocka M., Głowacka-Rutkowska A., Bednarek A., Borysowski J., Jończyk-Matysiak E., Łusiak-Szelachowska M., Weber-Dabrowska B., Bagińska N. (2018). Phage therapy: What have we learned?. Viruses.

[191] Venturini C., Fabjian A.P., Cy Lin R. (2019). Bacteriophage therapy for severe infections. Microbiol. Aust..

[192] Abedon S.T. (2017). Information phage therapy research should report. Pharmaceuticals.

